# The Active Brains Digital Intervention to Reduce Cognitive Decline in Older Adults: Protocol for a Feasibility Randomized Controlled Trial

**DOI:** 10.2196/18929

**Published:** 2020-11-20

**Authors:** Kirsten Ailsa Smith, Katherine Bradbury, Rosie Essery, Sebastien Pollet, Fiona Mowbray, Joanna Slodkowska-Barabasz, James Denison-Day, Victoria Hayter, Jo Kelly, Jane Somerville, Jin Zhang, Elisabeth Grey, Max Western, Anne E Ferrey, Adele Krusche, Beth Stuart, Nanette Mutrie, Sian Robinson, Guiqing Lily Yao, Gareth Griffiths, Louise Robinson, Martin Rossor, John Gallacher, Simon Griffin, Tony Kendrick, Shanaya Rathod, Bernard Gudgin, Rosemary Phillips, Tom Stokes, John Niven, Paul Little, Lucy Yardley

**Affiliations:** 1 Centre for Community and Clinical Applications of Health Psychology University of Southampton Southampton United Kingdom; 2 Primary Care and Population Sciences University of Southampton Southampton United Kingdom; 3 Department for Health University of Bath Bath United Kingdom; 4 Nuffield Department of Primary Care Health Sciences University of Oxford Oxford United Kingdom; 5 NIHR Oxford Biomedical Research Centre Oxford University Hospitals NHS Foundation Trust Oxford United Kingdom; 6 Physical Activity for Health Research Centre University of Edinburgh Edinburgh United Kingdom; 7 NIHR Newcastle Biomedical Research Centre Newcastle University and Newcastle upon Tyne NHS Foundation Trust Newcastle upon Tyne United Kingdom; 8 Southampton Clinical Trials Unit University of Southampton and University Hospital Southampton NHS Foundation Trust Southampton United Kingdom; 9 Institute of Population Health Sciences University of Newcastle Newcastle upon Tyne United Kingdom; 10 Dementia Research Centre University College London London United Kingdom; 11 Department of Psychiatry University of Oxford Oxford United Kingdom; 12 Department of Public Health and Primary Care University of Cambridge Cambridge United Kingdom; 13 Southern Health NHS Foundation Trust Southampton United Kingdom; 14 Public and Patient Involvement (PPI) representative University of Southampton Southampton United Kingdom; 15 School of Psychological Science University of Bristol Bristol United Kingdom

**Keywords:** telemedicine, dementia, internet-based intervention, geriatrics, feasibility studies, randomized controlled trial

## Abstract

**Background:**

Increasing physical activity, improving diet, and performing brain training exercises are associated with reduced cognitive decline in older adults.

**Objective:**

In this paper, we describe a feasibility trial of the Active Brains intervention, a web-based digital intervention developed to support older adults to make these 3 healthy behavior changes associated with improved cognitive health. The Active Brains trial is a randomized feasibility trial that will test how accessible, acceptable, and feasible the Active Brains intervention is and the effectiveness of the study procedures that we intend to use in the larger, main trial.

**Methods:**

In the randomized controlled trial (RCT), we use a parallel design. We will be conducting the intervention with 2 populations recruited through GP practices (family practices) in England from 2018 to 2019: older adults with signs of cognitive decline and older adults without any cognitive decline. Trial participants were randomly allocated to 1 of 3 study groups: usual care, the Active Brains intervention, or the Active Brains website plus brief support from a trained coach (over the phone or by email). The main outcomes are performance on cognitive tasks, quality of life (using EuroQol-5D 5 level), Instrumental Activities of Daily Living, and diagnoses of dementia. Secondary outcomes (including depression, enablement, and health care costs) and process measures (including qualitative interviews with participants and supporters) will also be collected. The trial has been approved by the National Health Service Research Ethics Committee (reference 17/SC/0463).

**Results:**

Results will be published in peer-reviewed journals, presented at conferences, and shared at public engagement events. Data collection was completed in May 2020, and the results will be reported in 2021.

**Conclusions:**

The findings of this study will help us to identify and make important changes to the website, the support received, or the study procedures before we progress to our main randomized phase III trial.

**Trial Registration:**

International Standard Randomized Controlled Trial Number 23758980; http://www.isrctn.com/ISRCTN23758980

**International Registered Report Identifier (IRRID):**

DERR1-10.2196/18929

## Introduction

### Background and Rationale

The prevalence of dementia is estimated to be between 5% and 7% among those aged 65 years and above [1,2]. Although the rates have fallen slightly in the United Kingdom over the past 20 years [2], the absolute number of cases is likely to increase owing to longer life spans [3]. Over the next 30 years, 10-15 million cases are predicted in Europe [3], and 132 million in the world by 2050 [4], with approximately 43% of prevalent cases needing high-level care (equivalent to nursing home care) [3]. It has been estimated that if interventions could delay both disease onset and progression by one year, there would be nearly 9.2 million fewer cases of the disease worldwide by 2050, almost all attributable to a reduction in persons needing a high level of care [3].

Cognitive impairment in the absence of dementia is common, but prevalence estimates vary considerably depending on the definition [5]. Mild cognitive impairment (MCI) is the most commonly used definition, conventionally defined as a deterioration in at least one nonmemory cognitive domain in addition to memory impairment, without severe functional impairment or loss of independence in Instrumental Activities of Daily Living [3,6]. The incidence of MCI is high in those aged above 65 years [7-9], ranging from 51 to 76.8 per 1000 person-years. There is not enough evidence yet that screening for MCI is either effective or cost-effective [10], but a systematic review of cohort studies suggests that between 5% and 10% of MCI cases progress into dementia annually [11]. However, defining cognitive impairment solely in terms of MCI misses a large group of individuals who are at a similar risk of developing dementia [12-14]. An alternative way of characterizing cognitive impairment is age-associated cognitive decline (AACD). AACD is described as 1 SD below normal cognitive functioning in any cognitive domain, with some investigators considering an additional criterion (self-report of a gradual decline in memory present for at least 6 months) [5]. The estimated prevalence of AACD depends on the number of cognitive domains assessed and whether additional criteria are used, which may explain the variable prevalence estimates—in one population-based study, up to 20% of those aged above 60 years had AACD using the simple criteria described by Ritchie et al [14] and as low as 1.4% in the UK Cognitive Functioning and Ageing Studies, where detailed criteria were used [5]. Progression to dementia is common no matter which definition is used, with rates almost comparable with that of MCI: 9% per annum in a population-based study (additional criterion not used) [14] and 10% in Cognitive Functioning and Ageing Studies (additional criterion used) [12,13]. Importantly, there is currently no diagnostic or treatment pathway for people with AACD. Given the scale of the problem, there are clearly insufficient resources on offer to help prevent cognitive decline or dementia in those with cognitive impairment or to prevent the development of cognitive impairment.

There is mounting evidence that healthy behaviors (particularly physical activity) and cognitive exercises improve cognitive functioning and activities of daily living, and a recent trial from other settings has demonstrated the potential effectiveness of combining healthy behavior and cognitive interventions [15].

### Healthy Behaviors: Physical Activity and Healthy Eating

Several large cohort studies and systematic reviews indicate that leisure time physical activity—even at moderate intensity levels—is protective, as is greater fish and fruit and vegetable consumption, with increased risks shown for obesity and high saturated fat intake [16-23]. The Caerphilly cohort is representative of the UK population and measures protective factors (not smoking, BMI levels, fruit and vegetable intake, physical activity, and moderate alcohol intake [22]), and after 30 years, it demonstrated markedly reduced risks of cognitive impairment (odds ratio [OR] for having 4 to 5 protective risk factors=0.36 and dementia OR=0.36). A trial of the Mediterranean diet for over four years demonstrated beneficial effects on cognitive decline among those with no cognitive deficits at baseline: changes in cognitive z-scores were 0.04 (95% CI −0.09 to 0.18) for the Mediterranean diet plus olive oil, 0.09 (−0.05 to 0.23 vs controls) for the Mediterranean diet plus nuts, and −0.17 (−0.32 to −0.01) for the control diet [24]. Interventions to increase physical activity are protective in the shorter term [25] with effects even at 6 months to a year on both cognitive decline, gray matter volume, and atrophy [26-28]. The population attributable risk (PAR) has been estimated for diabetes, hypertension, obesity, physical inactivity, depression, smoking, and low educational attainment, with the highest PAR found for physical inactivity (PAR 21.8%, 95% CI 6.1-37.7) [29].

### Cognitive Exercises

A systematic review of cognitive exercise trials [30] documented a strong effect size in cognitive performance (weighted mean difference=1.07; 95% CI 0.32 to 1.83; n=3194) with effects maintained after 2 years. A more recent systematic review of cognitive and memory training among those with MCI documented substantial heterogeneity of interventions [31] but a promising effect of cognitive exercises (effect sizes ranging from 0.10 to 1.21). The large Advanced Cognitive Training for Independent and Vital Elderly study investigated the effect of training in several cognitive domains (memory, reasoning, and processing speed) [32]; the change in reasoning (effect size=0.26) was particularly important, resulting in significantly less functional decline in activities of daily living, which was maintained over 5 years. Booster training produced additional improvement in reasoning performance (effect size=0.28) [33].

### Feasibility of Online Delivery

Although there is evidence that suggests healthy behavior changes can be protective against cognitive decline, behavioral interventions are complex and resource intensive, if delivered by purely face-to-face methods. In contrast, the internet is now used extensively and successfully by older people for self-management of health [33,34]. Although many individuals may benefit from using an intervention independently, the additional impact of behavioral facilitation may be important in helping initiate and maintain behavior change [35,36], and the effectiveness and cost-effectiveness of a more intensive intervention may vary with the risk of developing dementia.

#### Internet Use Among Older Adults

Recent UK government statistics demonstrate that the proportion of older adults using the internet is rapidly increasing. Among those aged 55 to 64 years, 88.3% reported recent (in the past 3 months) internet use. Although this proportion declines across age groups, this age group has demonstrated the fastest growth. Over the past 7 years, the proportion of adults aged 75 years and above reporting recent internet use has nearly doubled from 20% in 2011 to 47% in 2019 [37].

The research team has extensive experience of ensuring intervention engagement and accessibility to encompass a range of user preferences [38] and has experience of successfully supporting older patients, for example, exercises for dizziness [39], rehabilitation for stroke [40], and fall prevention [41]. The team can also draw upon existing experiences of developing suitable interventions to ensure engagement and accessibility for people of all ages and computer abilities, including those with lower health literacy [38,42].

#### Internet-Delivered Interventions Among a Cognitively Impaired Population

A large trial (n=2912) led by one of the research team’s coinvestigators (CB) [43] used a web-based cognitive training package in our target populations (Reasoning and problem-solving Cognitive Training [ReaCT] package) with improvements after 6 months in instrumental activities of daily living, reasoning, and verbal learning (standardized effect sizes of 0.15, 0.42, and 0.18, respectively). There were very similar effect sizes among those with cognitive impairment (AACD). This suggests that a web-based package is likely to be feasible and effective among those with cognitive decline. Although some people in our target population are currently not internet users, this proportion is rapidly declining [44]; therefore, the findings of this study will be relevant to the large majority of the older adult population in the future.

#### Intervening With Noncognitively Impaired Older Adults

Intervening with noncognitively impaired older adults may also prevent dementia. Although an intervention for noncognitively impaired older adults may be expected to have a smaller effect, data from the large ReaCT trial [43] suggest that this is not necessarily the case. Furthermore, the noncognitively impaired older adults are a substantially larger proportion of the population, so an intervention to target this group provides potential for helping many more of the older adult population. This population is also very concerned about developing dementia: 80% of those aged 50 years or above in a survey undertaken by Saga (n=9049) said they feared dementia, which was equivalent to the fear of cancer, and 84% feared dementia in their partners, which was more than they feared cancer in their partners [45]. Fear of dementia, the need to improve dementia knowledge, and having adequate support for behavioral change rather than simply being told what to do are likely to be major motivators toward changing health behaviors in an older adult general population group [46]. Thus, it is plausible that a well-designed and engaging behavioral intervention to help prevent cognitive decline and dementia will be both well-received and effective among an older adult general population sample and will be strongly supported by their partners.

### Objectives

The primary aim of this study (*the Active Brains study*—*feasibility trial* ISRCTN 23758980) is to assess the feasibility and acceptability of our trial procedures and of a web-based digital intervention (Active Brains), which helps support people in making healthy changes (physical activity, brain training, and diet) to maintain cognitive function and prevent cognitive decline.

#### Primary Research Objective

The primary outcome was to investigate the feasibility of collecting clinical outcomes and notes review data. This is built around our stop or go criteria for the proposed full RCT: the project will progress to a full RCT if 80% of the clinical outcome and notes review data from randomized participants are available for analysis. If the figures are 70%-80%, we will discuss our plans for appropriate mechanisms to increase the response rate with the trial steering committee. If less than 70% of the data are available, in negotiation with the funder, we will consider not proceeding to the main trial unless there is a clear and plausible plan to increase response rates or reduce missing data. The feasibility and resource requirements for recruitment will also affect the likelihood of progression to the main trial.

#### Secondary Research Objectives

The evaluation of feasibility and acceptability in terms of (1) suitability of recruitment screening methods; (2) acceptability of all trial procedures (eg, recruitment, randomization, study materials, follow-up); (3) recruitment and attrition rates; (4) acceptability of the digital intervention (uptake, usage, attrition, and qualitative process evaluation); (5) appropriateness of the human support module (uptake, adherence, number of sessions, and qualitative process evaluation); (6) suitability of all outcome measures; and (7) Health Economics analysis—the key resources to be collected—to inform the choice of quality of life instruments to be used in the full trial.

We will also explore the analysis of the characteristics of outcomes for power calculations to confirm the target sample size for the trial and preliminary estimates of change in relevant behaviors (based on automatically recorded intervention usage, eg, goals set and reviewed, scores on brain training games).

### Study Design

The randomized controlled study will use a parallel design. Participants will be divided into *cognitively impaired* and *noncognitively impaired* subgroups based on existing cognitive impairment (each subgroup is treated as a separate trial for randomization and reporting). A total of 180 participants from each group will be randomly allocated to 1 of 3 study groups (totaling 360 participants):

1. Usual care (60 participants from each group).
2. Access to the Active Brains intervention (60 participants from each group).
3. Access to the Active Brains intervention with flexible human support from a central support facilitator (60 participants from each group).

It is anticipated that the noncognitively impaired group will recruit more quickly than the cognitive impairment group, due to the low prevalence of MCI and AACD within the age group. Participants exceeding the allocated group size of 180 will not be randomized but will be offered to access the intervention (no support) as part of a cohort group.

## Methods

### Study Setting

Primary care recruitment will involve practice staff inviting patients from searches of UK practice lists based in England (GPs [General Practitioners] will screen practice lists to avoid inviting those who have an existing diagnosis of dementia and who are terminally ill or seriously mentally ill) between October 2018 and January 2019. Participants will also be recruited opportunistically during consultations with practitioners. Invitation letters, participant information sheets, reply slips (for those not interested to inform us why), and a *getting started* card with instructions on how to log on to the website to start the study will be sent from GP practices. Participants can also contact the research team directly if they have any questions. Following informed consent, GP practices will be asked to provide demographic data (gender, age, and postcode to help us establish the level of deprivation) for all participants who are invited to the study. We will compare the demographics of those invited but who do not participate in the study with recruited participants to examine any differences between these 2 groups. This will help the assessment of the likely generalizability of our findings.

### Eligibility Criteria

**Figure 1:**
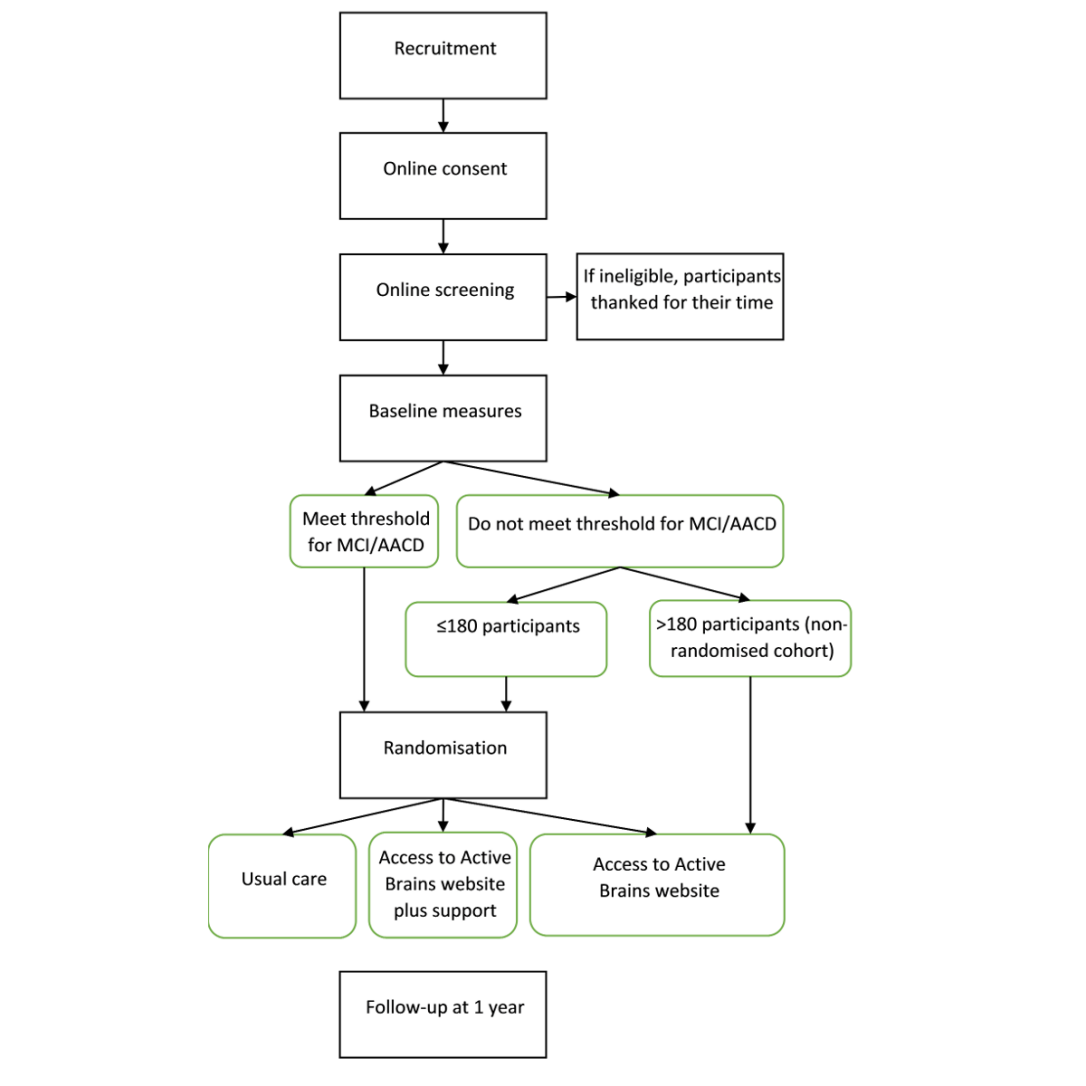
Randomized controlled trial and pilot study procedure flowchart. AACD: age-associated cognitive decline; MCI: mild cognitive impairment.

Figure 1 outlines the participant identification and screening procedures. Participants recruited through primary care will be screened to include only participants aged between 60 and 85 years and to exclude persons with a diagnosis of dementia, terminal illness, serious mental illness, or persons within the same household.

When participants first access the Active Brains study website, they will be asked to sign up with a username, password, and their unique ID code (provided on their study invitation letter). They are then presented with the information sheet and web-based consent form to complete. If they consent, they then answer a series of screening questions to ensure that they fit the study inclusion criteria (shown in Textbox 1). Age, dementia diagnosis, and the same household questions will be repeated in the web-based screening. Additionally, web-based screening will include willingness and ability to access the internet, the Godin Leisure Time Exercise Questionnaire (this questionnaire [47] will be used to screen highly physically active participants as it is a short and simple measure, collecting only sufficient data for screening participants and tailoring intervention content. The International Physical Activity Questionnaire [48] will be used to assess physical activity as an outcome measure as it has higher granularity and assesses sedentary behavior, which is targeted in the intervention), the Instrumental Activities of Daily Living Questionnaire (IADL) [49], and 4 cognitive tasks (short computer games that evaluate cognitive skills). If eligible, participants will be asked to complete baseline measures (Table 1).

**Inclusion and exclusion criteria for the Active Brains feasibility trial.**

**Step 1: General practitioner screening**

**Inclusion criteria**

- Between 60 and 85 years of age

**Exclusion criteria**

- Diagnosis of dementia
- Terminal Illness
- Serious mental illness

**Step 2: Web-based screening**

**Inclusion criteria**

- Able and willing to access the internet
- Cognitive impairment group: ≥1 SD below the norm on the Baddeley Reasoning Test [44]
- No cognitive impairment group: <1 SD below the norm (or above norm) on the Baddeley Reasoning Test [44]

**Exclusion criteria**

- High levels of leisure time physical activity (score ≥30 on Godin Leisure Time Exercise Questionnaire [47] (moderate or vigorous physical activity only)
- Another member of the household already participating in the study

**Table 1:** Outcome measures and times presented to each group for the Active Brains feasibility trial.

Measure		Time		Group			
		Baseline	12-month follow-up	Usual care	Active Brains intervention	Active Brains intervention and support from central facilitator	Nonrandomized cohort study participants (receiving the Active Brains intervention)
Sociodemographic data		✓^a^	—^b^	✓	✓	✓	✓
Clinical and behavioral data		✓	✓	✓	✓	✓	✓
Clinical measures (from notes review)		—	✓	✓	✓	✓	—
Cognitive Performance Tasks [50-53]		✓	✓	✓	✓	✓	✓
Instrumental Activities of Daily Living [49]		✓	✓	✓	✓	✓	✓
The Patient Enablement Scale [48]		—	✓	✓	✓	✓	✓
International Physical Activity Questionnaire [54] plus strength and balance items^c^		✓	✓	✓	✓	✓	✓
EQ-5D-5L^d^ [55]		✓	✓	✓	✓	✓	✓
EQ-5D-5L-proxy version 2 (for contact person completion if required) [55]		—	✓	✓	✓	✓	—
ICEpop CAPability measure [56]		✓	✓	✓	✓	✓	✓
Short-form Health Survey [57]		✓	✓	✓	✓	✓	✓
Dementia diagnosis (from notes review)		—	✓	✓	✓	✓	✓
Mortality		—	✓	✓	✓	✓	—
Health economic analysis of cost-effectiveness (from notes review)		—	✓	✓	✓	✓	—
Food Frequency Questionnaire [58]		✓	✓	✓	✓	✓	✓
Problematic Experiences of Therapy Scale [59]		—	✓	—	✓	✓	✓
Brief Geriatric Depression Scale [60,61]		✓	✓	✓	✓	✓	✓
Self-efficacy for exercise [62]		✓	✓	✓	✓	✓	✓
Medical Outcome social support Survey [63,64]		✓	—	✓	✓	✓	✓
Social Support for Exercise [65]		—	✓	✓	✓	✓	✓
Locus of Causation in Exercise [66]		✓	✓	✓	✓	✓	✓
Technology Acceptance Model Perceived Ease of Use scale [67-69]		—	✓	—	✓	✓	✓
Informant Questionnaire on Cognitive Decline in the Elderly–short form (if participants do not complete full measures) [70]		—	✓	—	✓	✓	—
**Objective patient data**							
	Use of the Active Brains intervention	—	—	—	✓	✓	✓
**Objective supporter data**							
	Supporters’ use of Active Brains intervention (throughout the study)	—	—	—	✓	✓	—
	Emails sent to participants throughout the study	—	—	—	✓	✓	—
**Qualitative data**							
	Interviews with patients about their experiences of the study and/or intervention	—	—	✓	✓	✓	—
	Interviews with Central Support Facilitators about their experiences of the study and intervention	—	—	✓	✓	✓	—

^a^✓: indicates yes.

^b^—: indicates no.

^c^“During the last 7 days, on how many days did you do activities or exercises to improve your strength and balance? Examples would be exercises such as standing on one leg, doing repeated sit to stands in a chair, lifting weights or heavy objects at home or at a gym or using resistance bands. Please only include time where you have purposefully decided to do these exercises for improving strength and balance.” Response requested: Number of days and number of hours or minutes on an average day.

^d^EQ-5D-5L: EuroQol-5D 5 level.

Subgroups of cognitively impaired participants will be identified by combinations of impairment in the Baddeley reasoning cognitive task, IADL, and memory cognitive task. Key subgroups are MCI, which will be defined as 1.5 SD below the norm in a nonmemory cognitive domain plus memory impairment [5,12,13], and AACD will be defined as 1 SD below the norm for the Baddeley Reasoning Test [50] and IADL.

### Assignment of Interventions

Simple randomization will be used to assign participants to intervention arms using the study software’s computer-generated random numbers. Randomization occurs online after a participant completes baseline measures, ensuring blind randomization. They will have an equal chance of being in each of the 3 groups. Once randomized, participants will be informed of their group allocation (they will also be emailed this information). If participants are in one of the treatment arms, they will be taken directly to the Active Brains intervention. These participants can then use the Active Brains intervention as much as they would like to over the course of the study. The central facilitator will be notified by email when a participant is randomized to the support arm of the trial, so that they know to expect to provide support to the participant in the coming weeks. Research staff will not be blinded to the participant group but the statistician and health economists conducting the analyses will be.

### Patient and Public Involvement

At the outset, the project recruited 2 older adult patient and public involvement (PPI) representatives to inform the development of the intervention and trial. They contributed to the development of the protocol and early intervention drafts. When intervention development began, it was determined that further PPI input would be essential to make the intervention and study materials engaging and easy to use. Three older adult representatives were recruited. Their input was invaluable in providing direction and feedback on the content and structure of the intervention (eg, the name, logo, content, order of pages) as well as the consent processes, interview topic guides, screening, and study measures (eg, feedback on unsuitable phrasing, resolving questionnaire burden).

During and after the trial, the PPI representatives will inform the discussion and interpretation of the trial outcomes and process evaluations and provide a patient perspective on resolving problems with recruitment, retention, participant support, and follow-up.

### Active Brains Intervention

The Active Brains intervention was developed to provide interactive tailored support to older adults in initiating and maintaining evidence-based behaviors that support cognitive and physical health using appropriate behavior change techniques. All intervention content has been iteratively developed with extensive input from the target user group to ensure that it is highly accessible and engaging. The Active Brains study website (containing the consent material and the study measures) is accessible to all participants, with only the intervention arm participants having access to the Active Brains intervention content. For the first 7 months, the intervention groups will have access to the Active Brains “Starter Section,” which provides support for users to initiate changes to their lifestyle in line with the intervention’s recommendations.

Within the first section of the Active Brains intervention, 3 modules will become available to users, released sequentially. Within these modules, users will have access to information addressing common concerns, instructions about recommended activities, guided goal setting and reviewing, personalized motivational feedback about their progress, and motivational reminder emails. The modules are as follows:

- Active Lives: a physical activity module that provides guidance on general physical activity, strength and balance exercises, and decreasing sedentary behavior.
- Brain Training: access to online *Brain Training* games.
- Eat for Health: guidance on healthy eating and increasing intake of foods beneficial for cognitive health.

After 6 months, users will be given access to the Active Brain “Booster Section,” providing further advice on habit formation and additional resources.

Textbox 2 summarizes the Active Brains intervention using the Template for Intervention Description and Replication (TIDieR) checklist [71]. For more details on the development of the Active Brains intervention, see the study by Essery et al (unpublished data, 2020).

**Description of the Active Brains intervention using the Template for Intervention Description and Replication checklist.**

1. Brief name

- Active Brains Study

2. Why

- An aging population places strains on health services: particularly with the increase in dementia. Several lifestyle changes have been found to help protect against cognitive decline. The aim of this study is to develop and test a digital behavior change intervention to help older adults increase their physical activity, improve their diet and practice cognitive 'Brain Training' activities to reduce cognitive decline.

3. What material?

- Assessment: All participants will complete web-based questionnaires and complete the four cognitive assessment games at 0 and 12 months
- Intervention Group: Participants in this group receive access to the Active Brains digital intervention.
- Intervention with support group: Participants in this group will receive the Active Brains digital intervention and additionally will be offered up to eight 10 minute support phone calls with a trained supporter.
- Control Group: Participants in this group will receive usual care from their GP and brief web-based advice about getting more active, improving diet and staying mentally active.
- The Active Brains Digital Intervention contains 4 sections described below.
- Month 0: Active Brains Starter Section. Access to 'Active Lives', including 'Strength and Balance', 'Taking Breaks from Sitting' and 'Getting Active'. These modules contain: Information addressing common concerns; Provision to order a step counter and guidance on using it; User stories modeling overcoming barriers; Instructions about obtaining social support; Suggestions for environment restructuring; Instructions about recommended activities (including Strength and Balance videos); Facilities to set, plan and review goals about their chosen activities; Tailored motivational feedback about their progress; Reminder emails to motivate them to continue with their activities and to revisit intervention content as appropriate.
- Month 1: Access to Brain Training. This module provides: Brief information about the rationale and evidence base for Brain Training tasks and how they are intended to work; User stories modeling overcoming barriers; Access to six web-based brain training games (via the existing PROTECT website) which they are encouraged to play between three and five times per week; facilities to set, plan and review goals about their chosen activities; tailored motivational feedback about their progress; reminder emails to motivate them to continue with their activities; Six additional games are made available to users during months 1-6
- Month 2: Access to Eat for Health. This module contains: Information on the benefits of healthy eating for cognitive and physical health; Information and techniques to allow them to make healthy changes to their existing eating behaviors (eg, increasing fruit and vegetable consumption, cutting down on processed foods); Facilities to set, plan and review goals about their chosen activities; User stories modeling overcoming barriers; Tailored motivational feedback about their progress; Reminder emails to motivate them to continue with their activities; Recipes
- Month 6: Transition to Active Brains Booster Section. This module contains: Tailored summary of their progress and engagement with the Starter Section content; Links to additional resources for additional support and to extend their progress with the behavioral changes made.
- The content was developed to support self-efficacy and autonomy. It used simple and accessible language, using persuasive rather than directive phrasing (ie, “you can” rather than “do”), and promoting guided choice rather than direction (eg, offering a range of ideas for goals).

4. What procedure

- Screening and randomization: Participants will complete web-based screening to ensure eligibility. Participants will then complete cognitive tasks and the Instrumental activities of daily living (IADL) questionnaire to determine work stream (with or without cognitive impairment). If the assigned work stream is full (capped at n=180), participants will be given access to Active Brains (no support) as part of a cohort study. Participants in each work stream will be randomized to one of the three groups. They will be informed of their group allocation on the website immediately.
- Intervention Group: Participants will receive full access to the Active Brains intervention. They will receive regular emails to remind them to access the intervention. They will be sent reminder emails when follow-up measures are available to complete.
- Intervention with Support Group: Participants will receive full access to the Active Brains intervention. Participants in this group will additionally be sent emails from an assigned supporter. They will be invited to book phone call appointments to support them with their use of the intervention.
- Control Group: Participants will receive basic information online about getting more active, improving diet and staying mentally active. They will be sent reminder emails when follow-up measures are available to complete.

5. Who provided

- Assessment: Participants will be invited to take part using a letter from their GP practice. They will access the screening and measures online.
- Intervention Group: Participants will use the intervention online.
- Intervention with Support group: Participants will additionally be provided with support from trained supported from the university of Southampton. These are members of the research team who have completed brief web-based training in the CARE approach. The CARE (Congratulate, Ask, Reassure, Encourage) approach is intended provide support to patients based on Self-Determination Theory. Supporters will also offer technical support.

6. How

- Intervention delivered entirely online.
- Follow-up procedures may include telephone and post.

7. Where

Intervention delivered entirely online, intended to be used in the participant's home.

8. When and how much

- Intervention Group: The intervention is divided into a 'Starter Section', lasting 6 months and a 'Booster' section provided at 6 months. The starter section unlocks 'Active Lives' at month 0, 'Brain Training' at month 1 and 'Eat for Health' at month 2. Participants will be encouraged to set goals and plans for each section and return to review the goals after one week. After 'Brain Training' unlocks, participants will be encouraged to set a goal to play the games 3-5 times a week until month 6, and then play for 3-5 times a week for a month every 3 months. Initially 6 games will be available with new games unlocking every month until 12 games are available. Participants will be guided to form healthy habits so that after 6 months they will be less reliant on the intervention. For participants with a BMI>30, a weight loss tool 'POWER' will be offered at month 6.
- Intervention with support group: In addition to the intervention, these participants will be offered three support phone calls at weeks 2, 6 and 10. They may request up to 8 phone calls in total.

9. Tailoring

- The intervention will tailor physical activity recommendations to self-rated Strength and Balance and current physical activity. Participants may then choose any or all of the three physical activity modules.
- Strength and balance exercises are tailored to self-rated strength and balance.
- The weight loss tool will only be offered to participants with a BMI>30.
- Goal review feedback will be tailored to prior performance (the past two review ratings for that module).

10. Modification

- The intervention will not be modified during the trial.

11. How well

- The intervention was created using the Person-Based Approach (PBA). Experts in digital behavior change oversaw the development. End users were consulted at all points of development and the intervention was iteratively refined to overcome anticipated barriers. The intervention was thoroughly tested by the research team to ensure that there were minimal problems with the software.

Participants will be encouraged to set and review goals every week when using Active Brains. When they unlock “Brain Training,” they will be encouraged to play the games 3 to 5 times per week for the first 6 months. The intervention will be available to the participants for a year. However, the Active Brains intervention aims to help participants establish healthy habits (eg, regular walking) so that participants are not dependent on the intervention in the long term—we anticipate that participants will not need to use the website every week for 6 months to gain benefits from the intervention. The level of engagement that is effective in improving outcomes will be investigated in the main trial.

### Central Facilitator Support

Patients in the group receiving support from a central facilitator (in addition to the website) will be offered a brief (10 min) telephone support call 2 weeks after they begin the study. In this support call, they will discuss the cognitive or lifestyle changes that they are planning to try. Patients will be offered 2 more support contacts by phone (up to 10 min) or email to support them in making behavioral changes.

Central facilitators will be people trained in the congratulate, ask, reassure, and encourage (CARE) approach to provide support to patients, which is based on Self-Determination Theory [72] and designed to promote autonomous motivation for making behavioral changes. This approach has been successful in previous trials [73].

### Usual Care

The comparison group will receive usual care from their GP practice, in addition to brief web-based advice about becoming more physically active, improving the diet, and staying mentally active.

### Outcome Measures

Table 1 presents a list of the measures and when they will be presented to participants. Participants will receive follow-up reminders by email, post, and telephone and by contacting a nominated contact person (optional). If no contact is successful, the contact person will be asked to help put the research team in touch with the participant or to complete 3 short questionnaires about the participant (EQ-5D-5L proxy version 2 [55], IADL [49], and Informant Questionnaire on Cognitive Decline in the Elderly [70]).

### Participant Timeline

Participants will register, be randomized, and complete baseline measures. Participants in the intervention arm will then be given access to the intervention, which they can use for one year. Participants in the support arms will be invited to have a support call at weeks 2, 6, and 10. A sample of participants will be invited to participate in a qualitative process interview at 6 months. All participants will be invited to complete outcome measures at 12 months.

### Sample Size

This is a feasibility trial, so it was not powered to measure patient outcomes. A sample size of 180 for each of the 2 subgroups was chosen for several reasons. As Active Brains is a digital intervention with multiple possible pathways through it, a sufficiently large group would be required to observe different patterns of usage. Fully web-based interventions are easy to recruit and manage larger numbers of participants, making this possible. The proposed fully powered trial would need to recruit over 20,000 patients, so implementation issues and trial processes (eg, managing multiple supporters) needed to be tested at scale. A total of 360 participants would provide robust evidence for the feasibility of the trial.

### Data Collection, Management, and Analysis

Outcome measures will be collected by using the software at registration and at one year. It will be held securely in a database accessible only by the data controller and members of the study team. Each participant will be assigned a unique study ID when they register with the website. Anonymous outcome data will be held in a separate write-only database to the personally identifiable data to ensure blinding and guard against modification.

Personally identifiable data will be held in accordance with the General Data Protection Regulation [74]. It will only be accessible to members of the study team throughout the study to monitor usage and provide support. The data will be analyzed by a statistician who has had no access to these data.

#### Statistical Analysis

The primary analysis will determine whether the feasibility trial has met the stop or go criteria and will therefore describe the completeness of the data in relation to the number of participants recruited.

Data for the secondary analyses will be explored descriptively and graphically for the other key feasibility outcomes, including intervention uptake, adherence, attrition, retention, and the number of participants recruited per practice. We will estimate the variability of proposed outcome measures and discuss any implications for the sample size for the full trial.

All participant data will be analyzed, including those who have withdrawn, unless the participant specifically requests for their data to be removed. All participants will be analyzed as randomized. The pattern and frequency of missing data will be explored descriptively in the feasibility context to determine whether there are whole instruments or items on instruments that participants opted not to complete and to inform the stop or go decision for the full trial.

The same outcomes will be assessed in a cohort study to explore whether it is feasible to recruit additional participants and collect data to inform future implementation in this way.

For the feasibility study, we will provide only descriptive estimates (ie, we will not be performing an inferential analysis to establish whether there are significant differences between groups).

#### Health Economic Analysis

At this feasibility stage, we aim to collect resource usage information associated with the intervention and explore and identify the likely changes in practice due to the intervention through questionnaires, case note review, and qualitative interviews. We plan to develop and refine the methods for collecting such information and to inform the choice of which instrument will be used to measure quality of life for the later planned, fully powered trial.

The study will explore both the National Health Service (NHS) and social service perspectives. All itemized resource usage will be costed using published information (eg, the Personal Social Services Research unit [75], the BNF [76]). Quality of life will be measured using EQ-5D [55], ICECAP [56], and SF-12 [57] at baseline and 12 months. We will apply the UK tariff to translate the questionnaire scores to utility scores. We aim to test the sensitivity and feasibility of these instruments in the study population, hence informing the choice of the instrument in the full trial.

The economic analyses of costs and quality of life will be mainly descriptive (with mean and standard deviation). We will correlate all utility scores with the planned primary outcome [50] to see the magnitude of sensitivities. The focus will be on the direction of correlation, spread, and confidence intervals.

Such information will allow us to investigate the most relevant resource use of information to be collected and inform choice of the quality of life instrument to be used in the definitive trial.

### Qualitative Process Analysis

There will be 2 qualitative process studies, one with participants and one with central support facilitators. Interviews in both these studies may be carried out at any time between 2 and 12 months after participants begin the study. Both of these studies will allow assessment of the acceptability and feasibility of the intervention and highlight any modifications to the intervention or study procedures that might be required before embarking on the later planned, fully powered trial.

#### Qualitative Process Study With Participants

We will interview 12 to 18 participants from each of the intervention arms, employing purposive sampling to ensure a diverse range of participants in terms of demographic and clinical profiles as well as website usage. Participants will be invited to participate by the research team and asked if they would be willing to take part in a telephone interview. They will complete a separate consent form (online) before taking part in an interview. During the interview, open-ended questions will be used to explore participants’ perceptions of the study, the website (if in one of the intervention groups), and the support they received from the central facilitator (if in the support group). Those in the control group will be asked about the brief advice they were given at baseline.

#### Qualitative Process Study With Central Support Facilitators

The second substudy will use face-to-face or telephone interviews to explore central support facilitators’ views of the study procedures, the website, the training they were provided, and the support they provided to patients (including perceptions of the CARE approach).

#### Analysis

Data from both qualitative process studies will be analyzed using inductive thematic analysis with interrater agreement reached between team members. The findings will be discussed and interpretations agreed between the coinvestigators (including PPI representatives).

### Monitoring Adverse Events

Serious adverse events (SAEs) will be reported by both participants, practice staff, and possibly by the central support facilitators who will have contact with participants during the study. It is not anticipated that SAEs will be related to this research. SAEs are defined as any untoward medical occurrence or effect that at any dose results in death, is life threatening, requires hospitalization or prolongation of existing hospitalization (excluding hospitalization for pre-existing conditions or planned procedures), results in persistent or significant disability or incapacity, is a congenital anomaly or birth defect, or results in other important medical events. Any SAE occurring to a research participant will be reported to the ethics committee where, based on the initial judgement made by the chief investigator or an agreed deputy, the event was related to the administration of any of the research procedures and was an unexpected occurrence. Nonserious adverse events will not be collected.

GP practices and the central support facilitation staff will inform the research team and/or the Clinical Trials Unit of any SAEs within 24 hours of them being aware of the event occurring. GP practices and central support facilitation staff will be provided with a standard operating procedure and a form for SAE reporting. Patients may also report SAEs. Reports of SAEs will be provided to the ethics committee within 15 days of the chief investigator becoming aware of the event. In addition to the chief investigator making the initial judgement about the SAE, all SAEs will also be sent to the trial steering committee for adjudication.

### Ethics

#### Informed Consent

Participants will be fully informed of the risks and benefits of the study and their right to withdraw at any time for any reason. Consent will be collected digitally before data collection.

#### Ethical Approvals

Ethical approval for the Active Brains study was obtained from the NHS Research Ethics Committee (IRAS ID 239448, REC number 17/SC/0463). Research and development approvals were obtained from relevant clinical research networks.

## Results

The results of this trial will be published in peer-reviewed journals and presented at conferences. Findings will be conveyed to the public through press releases and public engagement activities (eg, science fairs). Findings will be sent to all GP practices and participants who request them. Social media (eg, Twitter) will also be used to share publications and dissemination activities to the wider academic community and the general public. If found to be feasible, Active Brains will proceed to a full RCT.

Recruitment for this study was conducted from October 2018 to January 2019. All data were collected and the trial website was closed by May 2020. The analysis will be conducted in 2020, and results will be published in 2021. The prospective main trial will begin in late 2020.

## Discussion

The Active Brains feasibility trial has several strengths. First, the design of the intervention can be easily implemented at scale—this will be useful for the planned larger RCT and, if found to be effective, future dissemination to the public. Second, the development of the intervention used a person-based approach (combining theory, evidence, and primary qualitative research)—this has improved its chance of being acceptable and effective. *Active Brains* is the first web-based digital intervention to test this combination of evidence-based behavioral components for reducing cognitive decline (targeted physical activity, healthy eating, and brain training). This study will provide evidence for the feasibility of conducting a trial of an intervention similar to *Active Brains* and help us to improve upon the delivery of a larger trial. Limitations of the study are that this trial does not look at the long-term impact of healthy behavior change on cognitive health. This will be examined in a subsequent RCT.

## Appendix

Multimedia Appendix 1 Peer reviews of grant application.

## Abbreviations

AACD: age-associated cognitive decline
BRC: Biomedical Research Centre
CARE: congratulate, ask, reassure, and encourage
IADL: instrumental activities of daily living
MCI: mild cognitive impairment
NHS: National Health Service
NIHR: National Institute for Health Research
OR: odds ratio
PAR: population attributable risk
PPI: patient and public involvement
RCT: randomized controlled trial
ReaCT: Reasoning and problem-solving Cognitive Training
SAE: serious adverse event

## References

[1] Prince M, Bryce R, Albanese E, Wimo A, Ribeiro W, Ferri CP (2013). The global prevalence of dementia: a systematic review and metaanalysis. Alzheimers Dement.

[2] Matthews FE, Arthur A, Barnes LE, Bond J, Jagger C, Robinson L, Brayne C, Medical Research Council Cognitive Function and Ageing Collaboration (2013). A two-decade comparison of prevalence of dementia in individuals aged 65 years and older from three geographical areas of England: results of the cognitive function and ageing study I and II. Lancet.

[3] Brookmeyer R, Johnson E, Ziegler-Graham K, Arrighi HM (2007). Forecasting the global burden of Alzheimer's disease. Alzheimers Dement.

[4] (2017). Global Action Plan on the Public Health Response to Dementia 2017 - 2025. World Health Organisation.

[5] Stephan B, Matthews FE, McKeith IG, Bond J, Brayne C, Medical Research Council Cognitive Function and Aging Study (2007). Early cognitive change in the general population: how do different definitions work?. J Am Geriatr Soc.

[6] Petersen RC, Stevens J, Ganguli M, Tangalos EG, Cummings J, DeKosky ST (2001). Practice parameter: early detection of dementia: mild cognitive impairment (an evidence-based review). Report of the quality standards subcommittee of the American academy of neurology. Neurology.

[7] Ravaglia G, Forti P, Montesi F, Lucicesare A, Pisacane N, Rietti E, Dalmonte E, Bianchin M, Mecocci P (2008). Mild cognitive impairment: epidemiology and dementia risk in an elderly Italian population. J Am Geriatr Soc.

[8] Roberts R, Geda YE, Knopman DS, Cha RH, Pankratz VS, Boeve BF, Tangalos EG, Ivnik RJ, Rocca WA, Petersen RC (2012). The incidence of MCI differs by subtype and is higher in men: the Mayo clinic study of aging. Neurology.

[9] Luck T, Luppa M, Briel S, Riedel-Heller SG (2010). Incidence of mild cognitive impairment: a systematic review. Dement Geriatr Cogn Disord.

[10] Lin JS, O'Connor E, Rossom RC, Perdue LA, Burda BU, Thompson M, Eckstrom E (2020). Screening for Cognitive Impairment in Older Adults: An Evidence Update for the U.S. Preventive Services Task Force [Internet]. US Preventive Services Task Force Evidence Syntheses, formerly Systematic Evidence Reviews.

[11] Mitchell A, Shiri-Feshki M (2009). Rate of progression of mild cognitive impairment to dementia--meta-analysis of 41 robust inception cohort studies. Acta Psychiatr Scand.

[12] Stephan BC, Minett T, Terrera GM, Matthews FE, Brayne C (2015). Dementia prediction for people with stroke in populations: is mild cognitive impairment a useful concept?. Age Ageing.

[13] Stephan BC, Brayne C, McKeith IG, Bond J, Matthews FE, Medical Research Council Cognitive Function and Ageing Study (2008). Mild cognitive impairment in the older population: who is missed and does it matter?. Int J Geriatr Psychiatry.

[14] Ritchie K, Artero S, Touchon J (2001). Classification criteria for mild cognitive impairment: a population-based validation study. Neurology.

[15] Ngandu T, Lehtisalo J, Solomon A, Levälahti E, Ahtiluoto S, Antikainen R, Bäckman L, Hänninen T, Jula A, Laatikainen T, Lindström J, Mangialasche F, Paajanen T, Pajala S, Peltonen M, Rauramaa R, Stigsdotter-Neely A, Strandberg T, Tuomilehto J, Soininen H, Kivipelto M (2015). A 2 year multidomain intervention of diet, exercise, cognitive training, and vascular risk monitoring versus control to prevent cognitive decline in at-risk elderly people (FINGER): a randomised controlled trial. Lancet.

[16] Lee Y, Back JH, Kim J, Kim S, Na DL, Cheong H, Hong CH, Kim YG (2010). Systematic review of health behavioral risks and cognitive health in older adults. Int Psychogeriatr.

[17] Lafortune L, Martin S, Kelly S, Kuhn I, Remes O, Cowan A, Brayne C (2016). Behavioural risk factors in mid-life associated with successful ageing, disability, dementia and frailty in later life: a rapid systematic review. PLoS One.

[18] Gelber RP, Petrovitch H, Masaki KH, Abbott RD, Ross GW, Launer LJ, White LR (2012). Lifestyle and the risk of dementia in Japanese-American men. J Am Geriatr Soc.

[19] Tangney CC, Li H, Wang Y, Barnes L, Schneider JA, Bennett DA, Morris MC (2014). Relation of DASH- and Mediterranean-like dietary patterns to cognitive decline in older persons. Neurology.

[20] Singh-Manoux A, Czernichow S, Elbaz A, Dugravot A, Sabia S, Hagger-Johnson G, Kaffashian S, Zins M, Brunner EJ, Nabi H, Kivimäki M (2012). Obesity phenotypes in midlife and cognition in early old age: the Whitehall II cohort study. Neurology.

[21] Hagger-Johnson G, Sabia S, Brunner EJ, Shipley M, Bobak M, Marmot M, Kivimaki M, Singh-Manoux A (2013). Combined impact of smoking and heavy alcohol use on cognitive decline in early old age: Whitehall II prospective cohort study. Br J Psychiatry.

[22] Elwood P, Galante J, Pickering J, Palmer S, Bayer A, Ben-Shlomo Y, Longley M, Gallacher J (2013). Healthy lifestyles reduce the incidence of chronic diseases and dementia: evidence from the Caerphilly cohort study. PLoS One.

[23] Eskelinen MH, Ngandu T, Helkala E, Tuomilehto J, Nissinen A, Soininen H, Kivipelto M (2008). Fat intake at midlife and cognitive impairment later in life: a population-based CAIDE study. Int J Geriatr Psychiatry.

[24] Valls-Pedret C, Sala-Vila A, Serra-Mir M, Corella D, de la Torre R, Martínez-González MA, Martínez-Lapiscina EH, Fitó M, Pérez-Heras A, Salas-Salvadó J, Estruch R, Ros E (2015). Mediterranean diet and age-related cognitive decline: a randomized clinical trial. JAMA Intern Med.

[25] Buchman A, Boyle P, Yu L, Shah R, Wilson R, Bennett D (2012). Total daily physical activity and the risk of AD and cognitive decline in older adults. Neurology.

[26] Ruscheweyh R, Willemer C, Krüger K, Duning T, Warnecke T, Sommer J, Völker K, Ho H, Mooren F, Knecht S, Flöel A (2011). Physical activity and memory functions: an interventional study. Neurobiol Aging.

[27] Gow AJ, Bastin ME, Maniega SM, Hernández MC, Morris Z, Murray C, Royle NA, Starr JM, Deary IJ, Wardlaw JM (2012). Neuroprotective lifestyles and the aging brain: activity, atrophy, and white matter integrity. Neurology.

[28] Ahlskog JE, Geda YE, Graff-Radford NR, Petersen RC (2011). Physical exercise as a preventive or disease-modifying treatment of dementia and brain aging. Mayo Clin Proc.

[29] Norton S, Matthews FE, Barnes DE, Yaffe K, Brayne C (2014). Potential for primary prevention of Alzheimer's disease: an analysis of population-based data. Lancet Neurol.

[30] Valenzuela M, Sachdev P (2009). Can cognitive exercise prevent the onset of dementia? Systematic review of randomized clinical trials with longitudinal follow-up. Am J Geriatr Psychiatry.

[31] Gates NJ, Sachdev PS, Fiatarone Singh MA, Valenzuela M (2011). Cognitive and memory training in adults at risk of dementia: a systematic review. BMC Geriatr.

[32] Willis SL, Tennstedt SL, Marsiske M, Ball K, Elias J, Koepke KM, Morris JN, Rebok GW, Unverzagt FW, Stoddard AM, Wright E, ACTIVE Study Group FT (2006). Long-term effects of cognitive training on everyday functional outcomes in older adults. J Am Med Assoc.

[33] Aalbers T, Baars M, Rikkert MO (2011). Characteristics of effective internet-mediated interventions to change lifestyle in people aged 50 and older: a systematic review. Ageing Res Rev.

[34] Stellefson M, Chaney B, Barry AE, Chavarria E, Tennant B, Walsh-Childers K, Sriram P, Zagora J (2013). Web 2.0 chronic disease self-management for older adults: a systematic review. J Med Internet Res.

[35] Dennison L, Morrison L, Lloyd S, Phillips D, Stuart B, Williams S, Bradbury K, Roderick P, Murray E, Michie S, Little P, Yardley L (2014). Does brief telephone support improve engagement with a web-based weight management intervention? Randomized controlled trial. J Med Internet Res.

[36] Riper H, Blankers M, Hadiwijaya H, Cunningham J, Clarke S, Wiers R, Ebert D, Cuijpers P (2014). Effectiveness of guided and unguided low-intensity internet interventions for adult alcohol misuse: a meta-analysis. PLoS One.

[37] (2019). Internet Users, UK. Office for National Statistics.

[38] Yardley L, Morrison LG, Andreou P, Joseph J, Little P (2010). Understanding reactions to an internet-delivered health-care intervention: accommodating user preferences for information provision. BMC Med Inform Decis Mak.

[39] Essery R, Kirby S, Geraghty AW, Andersson G, Carlbring P, Bronstein A, Little P, Yardley L (2015). The development of balance retraining: an online intervention for dizziness in adults aged 50 years and older. Am J Audiol.

[40] Meagher C (2020). Feasibility randomised control trial of LifeCIT, a web-based support programme for constraint induced therapy (CIT) following stroke compared with usual care. Physical Therapy.

[41] Yardley L, Nyman SR (2007). Internet provision of tailored advice on falls prevention activities for older people: a randomized controlled evaluation. Health Promot Int.

[42] Rowsell A, Muller I, Murray E, Little P, Byrne CD, Ganahl K, Müller G, Gibney S, Lyles CR, Lucas A, Nutbeam D, Yardley L (2015). Views of people with high and low levels of health literacy about a digital intervention to promote physical activity for diabetes: a qualitative study in five countries. J Med Internet Res.

[43] Corbett A, Owen A, Hampshire A, Grahn J, Stenton R, Dajani S, Burns A, Howard R, Williams N, Williams G, Ballard C (2015). The effect of an online cognitive training package in healthy older adults: an online randomized controlled trial. J Am Med Dir Assoc.

[44] (2016). Internet users in the UK. Office for National Statistics.

[45] (2013). Over 50s Fear Dementia More Than Cancer. Saga Magazine.

[46] Kim S, Sargent-Cox KA, Anstey KJ (2015). A qualitative study of older and middle-aged adults' perception and attitudes towards dementia and dementia risk reduction. J Adv Nurs.

[47] Godin G, Shephard RJ (1985). A simple method to assess exercise behavior in the community. Can J Appl Sport Sci.

[48] Little P, Lewith G, Webley F, Evans M, Beattie A, Middleton K, Barnett J, Ballard K, Oxford F, Smith P, Yardley L, Hollinghurst S, Sharp D (2008). Randomised controlled trial of Alexander technique lessons, exercise, and massage (ATEAM) for chronic and recurrent back pain. Br Med J.

[49] Landi F, Tua E, Onder G, Carrara B, Sgadari A, Rinaldi C, Gambassi G, Lattanzio F, Bernabei R, SILVERNET-HC Study Group of Bergamo (2000). Minimum data set for home care: a valid instrument to assess frail older people living in the community. Med Care.

[50] Baddeley AD (2013). A 3 min reasoning test based on grammatical transformation. Psychon Sci.

[51] Wild K, Howieson D, Webbe F, Seelye A, Kaye J (2008). Status of computerized cognitive testing in aging: a systematic review. Alzheimers Dement.

[52] Conklin H, Curtis CE, Katsanis J, Iacono WG (2000). Verbal working memory impairment in schizophrenia patients and their first-degree relatives: evidence from the digit span task. Am J Psychiatry.

[53] Owen A, Beksinska M, James M, Leigh P, Summers B, Marsden C, Quinn N, Sahakian B, Robbins T (1993). Visuospatial memory deficits at different stages of Parkinson's disease. Neuropsychologia.

[54] Hurtig-Wennlöf S, Hagströmer M, Olsson LA (2010). The international physical activity questionnaire modified for the elderly: aspects of validity and feasibility. Public Health Nutr.

[55] Pickard AS, Wilke CT, Lin H, Lloyd A (2007). Health utilities using the EQ-5D in studies of cancer. Pharmacoeconomics.

[56] Coast J, Flynn TN, Natarajan L, Sproston K, Lewis J, Louviere JJ, Peters TJ (2008). Valuing the ICECAP capability index for older people. Soc Sci Med.

[57] Ware JE, Sherbourne CD (1992). The MOS 36-item short-form health survey (SF-36). I. Conceptual framework and item selection. Med Care.

[58] Little P, Barnett J, Margetts B, Kinmonth AL, Gabbay J, Thompson R, Warm D, Warwick H, Wooton S (1999). The validity of dietary assessment in general practice. J Epidemiol Community Health.

[59] Kirby S, Donovan-Hall M, Yardley L (2014). Measuring barriers to adherence: validation of the problematic experiences of therapy scale. Disabil Rehabil.

[60] Lesher EL, Berryhill JS (1994). Validation of the geriatric depression scale--short form among inpatients. J Clin Psychol.

[61] Almeida OP, Almeida SA (1999). Short versions of the geriatric depression scale: a study of their validity for the diagnosis of a major depressive episode according to ICD-10 and DSM-IV. Int J Geriatr Psychiatry.

[62] Resnick B, Jenkins LS (2000). Testing the reliability and validity of the self-efficacy for exercise scale. Nurs Res.

[63] Sherbourne CD, Stewart AL (1991). The MOS social support survey. Soc Sci Med.

[64] Moser A, Stuck AE, Silliman RA, Ganz PA, Clough-Gorr KM (2012). The eight-item modified medical outcomes study social support survey: psychometric evaluation showed excellent performance. J Clin Epidemiol.

[65] Sallis JF, Grossman RM, Pinski RB, Patterson TL, Nader PR (1987). The development of scales to measure social support for diet and exercise behaviors. Prev Med.

[66] Markland D, Hardy L (1997). On the factorial and construct validity of the intrinsic motivation inventory: conceptual and operational concerns. Res Q Exerc Sport.

[67] Venkatesh V, Davis FD (2000). A theoretical extension of the technology acceptance model: four longitudinal field studies. Manag Sci.

[68] Davis FD, Bagozzi RP, Warshaw PR (1989). User acceptance of computer technology: a comparison of two theoretical models. Manag Sci.

[69] Davis FD (1989). Perceived usefulness, perceived ease of use, and user acceptance of information technology. MIS Q.

[70] Jorm AF (1994). A short form of the informant questionnaire on cognitive decline in the elderly (IQCODE): development and cross-validation. Psychol Med.

[71] Hoffmann TC, Glasziou PP, Boutron I, Milne R, Perera R, Moher D, Altman DG, Barbour V, Macdonald H, Johnston M, Lamb SE, Dixon-Woods M, McCulloch P, Wyatt JC, Chan A, Michie S (2014). Better reporting of interventions: template for intervention description and replication (TIDieR) checklist and guide. Br Med J.

[72] Ryan RM, Deci EL (2000). Self-determination theory and the facilitation of intrinsic motivation, social development, and well-being. Am Psychol.

[73] Smith E, Bradbury K, Scott L, Steele M, Little P, Yardley L (2017). Providing online weight management in primary care: a mixed methods process evaluation of healthcare practitioners' experiences of using and supporting patients using POWER. Implement Sci.

[74] Voigt P (2017). The EU General Data Protection Regulation (GDPR): A Practical Guide.

[75] Curtis L (2018). Unit Costs of Health and Social Care 2018. Personal Social Services Research Unit.

[76] (2019). BNF British National Formulary - NICE.

