# Supplementary material for: The Active Brains Digital Intervention to Reduce Cognitive Decline in Older Adults: Protocol for a Feasibility Randomized Controlled Trial
Source: JMIR Res Protoc. 2020 Nov 20;9(11):e18929. doi: 10.2196/18929 (PMC7718093; doi:10.2196/18929)
Supplement: Multimedia Appendix 1 [file resprot_v9i11e18929_app1.pdf]

# NATIONAL INSTITUTE FOR HEALTH RESEARCH

# PROGRAMME GRANTS FOR APPLIED RESEARCH

## Feedback on the Stage 1 Application

| | |
|---|---|
| **Stage 1 Reference Number** | RP-PG-0615-10007 |
| **Lead Applicant** | Professor Paul Little |

As indicated in the Stage 2 guidance for applicants, all Stage 1 applications were assessed by an expert sub-panel of the NIHR Programme Grants for Applied Research Main Selection Panel using defined criteria. Those applications which best met the selection criteria were shortlisted and then sent for a comprehensive methodological review (i.e., from the perspectives of statistics, health economics, health psychology, Patient and Public Involvement (PPI) and qualitative methods) before applicants were invited to submit a Stage 2 application.

Please find below the feedback from the Stage 1 sub-panel's assessment of the application (incorporating comments from a PPI perspective). Anonymised versions of the statistics, health economics, health psychology and qualitative methods assessments can be found on subsequent pages.

Applicants are expected to address both the main and methodological feedback detailed about their Stage 1 application (and explain how their Stage 2 application has changed as a result of the feedback received) using the relevant question in the 'Case for Support – Part 2' section of the Stage 2 application form.

- The case for the proposal needed strengthening – it would be important to highlight the current and ongoing literature that the proposal was drawing upon to design the programme of research (i.e. some evidence that the approach proposed might be effective).
- The proposal's value for money was questioned. Specifically, further justification must be given for including an extra trial in non-cognitively impaired people and five year follow up - would this add value to a three arm trial in a cognitively impaired population?
- It would be important to describe anticipated problems in combining cognitive and lifestyle interventions and how the development work would tackle these specific issues.
- The use of computers in patients with potential cognitive impairment required justification.
- It should be clarified how findings would translate into health service benefit within five years of the programme given the proposed 5 years follow up.
- It was queried whether there might be scope for a clinically relevant outcome in the trials proposed.
- It should be clarified how the potential for benefit in a population would be determined with recruitment from pre-existing cohorts.
- It should be clarified how the inclusion criteria would be operationalised in assessing patients for delivery of the intervention in routine clinical service.

- Some members of the subpanel expressed concerns about the focus of the trials, considering them to be overly focussed on the technology aspects rather than on the patients.
- WS 2 must be better specified as it lacked detail (for example, more about the previous research in the general population, what the actual inclusion criteria would be, how the intervention might differ etc.).
- The overall arrangements for patient and public involvement (PPI) and the quality of the plain English summary were welcomed.
- Whilst NHS Treatment Costs and NHS Support Costs have been detailed in your finance form, we would encourage you to check the allocation of costs and engage with your local Network and your Trust's R&D or Finance office. Furthermore, we strongly recommend that you consult the latest finance guidance from the Department of Health (Attributing the costs of health and social care Research and Development (AcoRD)) when preparing your Stage 2 application.
- It is noted that background intellectual property (IP) will be utilised in the proposed programme in the form of (but not limited to) web based cognitive exercises and LifeGuide Software. When preparing your Stage 2 application, please provide further information about the use of this background IP, specifically: (a) whether any permissions/licenses to use and, if necessary, modify this background intellectual property in the proposed programme of research are required (and thus will be sought), and (b) the implications of any restrictions imposed by the owners and/or funders that would impact upon the future roll-out within the NHS.
- Intellectual property (IP) will be developed during the course of the programme including web-based intervention software and the training and intervention package for facilitators. The NIHR is particularly interested to ensure that thorough consideration is given to the available options to realise patient benefits arising from research it funds. It is therefore important that any arising IP (soft or hard) is sufficiently protected and we recommend that you discuss this matter further with your local IP advisor or Technology Transfer Office (TTO), bearing in mind that it is our expectation that all foreground IP will vest in the host NHS Trust.

National Institute for Health Research

# Programme Grants for Applied Research
# Methodology review form

| | |
|---|---|
| **Reference number** | RP-PG-0615-10007 |
| **Application Title** | REducing and preventing COgnitive impairment iN older age groups (the RECON Programme) |
| **Lead Applicant** | Professor Paul Little |
| **Lead Applicant Organisation** | Southern Health NHS Foundation Trust |
| **Total Amount Requested** | 3,431,585.00 |
| **Reviewer** | Information Redacted |

## Qualitative Assessment

| 1. Please comment on the quality of any qualitative work in the proposal:<br><br>The particular strengths of the qualitative work proposed |
|---|
| The qualitative work proposed is appropriate in methodology, method and position within the programme. |

| What you consider to be serious concerns that undermine the scientific quality of the qualitative work proposed |
|---|
| None |

| Other less serious concerns |
|---|
| In the full proposal, the purpose of the initial qualitative data collection needs to be made clearer, particularly how it will inform other aspects of the study. |

| 2. Comments on broader aspects of the proposal:<br><br>Particular strengths of the proposal? |
|---|
| Ambitious but if successful has the potential to change how people approach getting old |

| 3. Any other comments |
|---|
| None |

National Institute for Health Research

# Programme Grants for Applied Research
# Methodology review form

| | |
|---|---|
| **Reference number** | RP-PG-0615-10007 |
| **Application Title** | REducing and preventing COgnitive impairment iN older age groups (the RECON Programme) |
| **Lead Applicant** | Professor Paul Little |
| **Lead Applicant Organisation** | Southern Health NHS Foundation Trust |
| **Total Amount Requested** | 3,431,585.00 |
| **Reviewer** | Information Redacted |

## Statistics Assessment

| 1. Please comment on the quality of any statistics in the proposal:<br><br>The particular strengths of the statistical work proposed |
|---|
| A trials unit is on board with this application and two statisticians, one with considerable trials experience. Some considerable thought has been given to the sample size calculations in this application. |

| What you consider to be serious concerns that undermine the scientific quality of the statistical work proposed |
|---|
| None |

| Other less serious concerns |
|---|
| There isn't any detail of the statistical apporaches to be used in WS 1.1., and no PICOS detailed. I think the feasiblity trial needs a bit more thikning about - the list of issues to be addressed looks quite standard with very little detail. While some considerable thought has been given to the sample size calculations for WS4 I think the rationale for the approach (ie which groups to compare with which, conceptualisation of "success") needs to be stronger. Would 90% power be considered rather than 80%? I could noty replicate the final sample size of 10940 for the developmewnt of dementia sample size - this did not seem to match the number in each group of 1094. Perhaps I have misunderstood something - if so, it needs explaining more clearly. |

| 2. Comments on broader aspects of the proposal:<br><br>Particular strengths of the proposal? |
|---|
| Important issue, good team, their previous work that builds towards this. |

| 3. Any other comments |
|---|
| I could not find any references in the proposal so was not sure what was meant by eg "theoretical modelling" and I felt there could have been a stronger theoretical underpinning for the work. Some parts of the proposal seemed hastily written eg first part of section 1.4 repeated parts of 1.3. I wasn't fully convinced about the need for WS2 and think this needs a stronger justification  or removing. |

National Institute for Health Research

# Programme Grants for Applied Research
# Methodology review form

| | |
|---|---|
| **Reference number** | RP-PG-0615-10007 |
| **Application Title** | REducing and preventing COgnitive impairment iN older age groups (the RECON Programme) |
| **Lead Applicant** | Professor Paul Little |
| **Lead Applicant Organisation** | Southern Health NHS Foundation Trust |
| **Total Amount Requested** | 3,431,585.00 |
| **Reviewer** | Information Redacted |

## Health Psychology/Behavioural Assessment

| 1. Please comment on the quality of any health psychology/behavioural work in the proposal:<br><br>The particular strengths of the health psychology/behavioural work proposed |
|---|
| This is strong group who bring with them extensive experience and expertise in the development of internet based intervetions for different groups of patients. They describe sensible approaches for developing and refining the intervention that are based on apropriate frameworks of behaviour change. They have also done much of the preparation work here, having undertaken a systematic review on cognitive exercises and having developed an effective healthy lifestyle packages to provide behavioural support that they will adapt to this age group. |

| What you consider to be serious concerns that undermine the scientific quality of the health psychology/behavioural work proposed |
|---|
| I do not have any serious concerns, but there are a number of issues that the authors might want to consider more fully. These are described below. |

| Other less serious concerns |
|---|
| It was not entirely clear from the description of the intervention how frequently older people were expected to engage with the internet based intervention over the year. In other words, do we know the dose at which the intervention becomes effective? Is it possible to answer this question as part of the programme?<br>If daily engagement will be required, then the intervention developers may need to work quite hard on encouraging habit formation. The extent to which the intervention is engaging will obviously be part of the story here, but encouraging use in the first couple of weeks may need to be a more active process. Then, maintenance of use will be required. If this intervention were being delivered as part of routine primary care practice then one might envisage that the intervention is prescribed by a GP and the patient would visit the GP for a follow-up every six months when monitoring and feedback could take place and a new prescription for the intervention provided. However, it is not clear what will be support the patient to continue to adhere to the intervention without this. I acknowledge that more is done to encourage uptake and maintenance in the second intervention group, but the two interventions do appear to be at two extremes.<br>This is linked to my second concern - how such an intervention would be delivered as part of routine practice. Is it anticipated that patients will be invited through letters from GP surgeries?<br>If, after the first year, only one of the interventions appears to be effective in comparison with the control group, is there any scope for completing the follow-up based on only two groups? This may lead to a reduction in the resources required. Indeed, could this decision be made even earlier if the feasibility study examined uptake and then a larger pilot study investigated which of the interventions was mot effective in the first year. This could then be used as a basis for design of the main trial. |

| 2. Comments on broader aspects of the proposal |
|---|
| Little detail given about the role of applicants on the bid.<br>Good to see stopping rules set out.<br>I calculated that 20 participants per group across 6 groups was 120 participants for the feasibility study. The authors give 180. The authors seem to base their calculations on there being 5 groups, but I struggled to work our what these five groups were - could this be explained more clearly.<br>Why the cut-off of 75 years of age for inclusion. Can people aged 75+ not benefit from this? |

| 3. Any other comments |
|---|
| |

National Institute for Health Research

# Programme Grants for Applied Research
# Methodology review form

| | |
|---|---|
| **Reference number** | RP-PG-0615-10007 |
| **Application Title** | REducing and preventing COgnitive impairment iN older age groups (the RECON Programme) |
| **Lead Applicant** | Professor Paul Little |
| **Lead Applicant Organisation** | Southern Health NHS Foundation Trust |
| **Total Amount Requested** | 3,431,585.00 |
| **Reviewer** | Information Redacted |

## Health Economics Assessment

| 1. Please comment on the quality of any health economics in the proposal:<br><br>The particular strengths of the health economic work proposed |
|---|
| The aim is to develop and test efficient internet-supported lifestyle/cognitive exercises to reduce cognitive decline among older age adults. Two populations (each of 10,000+ participants) will be studies in two workstreams: (1) Mild Cognitive Impairment (MCI) and Age Associated Cognitive decline (AACD), and (2) no cognitative impairment. Participants will be randomly allocated to brief advice, internet package or facilitated internet package (by email/phone).<br>The proposal is ambitious, and the team is experienced and appropriately constructed. |

| What you consider to be serious concerns that undermine the scientific quality of the health economic work proposed |
|---|
| Small effect sizes are mooted for which very large sample sizes are required. The applicants should provide an explanation of what these effect sizes mean in physical terms (what will be the absolute improvement of CF, ADL, QALYs and why they are worthwhile)?<br>The trial size is predicated on showing an effect upon cognitive functioning at 5 years (with loss to follow up). Powering to one year appears to dramatically reduce the sample size (and cost of the study?). Using AUC or repeated measures approaches might be more efficient than a simple 5 year endpoint, for a study that might prove ineffective at one year.<br>As currently described the collection of health economic data is not fit for purpose given the long gaps (baseline, one year, five years) the method (questionnaire - recall?) proposed and the participants (in cognitive decline). For example is it plausible that participants with cognitive decline can recall their informal and formal social care and employment over such timescales. How will NHS resource use be identified and attributed?<br>Given that only 60% of subjects are anticipated to complete 5 years, yearly QALY measurement might be a minimum. A better strategy for economic modelling might be to get high quality one year data (with multiple points within the first year). How will follow-up visits be made (in person or by email)? What steps will be taken to minimise missing data during follow-up? |

| Other less serious concerns |
|---|
| Although the PPI is appropriate it is unclear how it has informed the proposal beyond consultation with the people and groups described. |

| 2. Comments on broader aspects of the proposal |
|---|
| ... |

| 3. Any other comments |
|---|
| ... |
